# Коррекция общего кальция в крови на альбумин: есть ли целесообразность?

**DOI:** 10.14341/probl13503

**Published:** 2024-11-13

**Authors:** Е. О. Мамедова, О. О. Голоунина, Ж. Е. Белая

**Affiliations:** Национальный медицинский исследовательский центр эндокринологии; Национальный медицинский исследовательский центр эндокринологии; Национальный медицинский исследовательский центр эндокринологии

**Keywords:** кальций, общий кальций, ионизированный кальций, альбумин, гипокальциемия, гиперкальциемия, хроническая болезнь почек, первичный гиперпаратиреоз, критические состояния

## Abstract

Кальций является самым распространенным минералом в организме человека. Около 99% кальция депонируется в  костной ткани в виде солей гидроксиапатита и только 1% находится во внутриклеточном и внеклеточном пространстве. Биологически активным является ионизированный кальци, составляющий примерно 50% от общего количества циркулирующего кальция, остальная часть связана с белками плазмы (40%, при этом в 90% случаев это альбумин, в 10% — глобулины) или находится в комплексе с анионами (10%), такими как цитрат, лактат, бикарбонат, фосфат. Гипо- и гиперкальциемия — частые состояния, с которыми сталкиваются врачи различных специальностей. Первичный гиперпаратиреоз и злокачественные новообразования являются наиболее распространенными причинами гиперкальциемии, тогда как гипокальциемия чаще всего обусловлена гипопаратиреозом, мальабсорбцией, дефицитом витамина D или хронической болезнью почек. Интерпретация результатов определения уровня кальция в крови влияет на постановку правильного диагноза, необходимость назначения дальнейшего обследования, выбор лечения. Определение уровня ионизированного кальция считается более точным показателем истинного статуса кальция по сравнению с концентрацией общего кальция, однако его измерение затруднено ввиду строгих преаналитических и аналитических требований. В середине 1970-х годов появились уравнения корректировки содержания кальция, широко используемые в настоящее время. Однако некоторые исследования позволили усомниться в достаточной надежности и чувствительности соответствующих корректировочных формул. Диагностическая точность широко используемых формул коррекции в некоторых клинических ситуациях отличается от диагностической точности нескорректированного общего кальция в худшую сторону. В обзоре обсуждается история возникновения формул коррекции общего кальция на альбумин, факторы, влияющие на результат коррекции, а также ее целесообразность при различных состояниях.

## Abstract (en)

Calcium is the most abundant mineral in the human body. About 99% of calcium is deposited in the bones in the form of hydroxyapatite and only 1% is located in the intracellular and extracellular fluid. Ionized calcium, which makes up about 50% of the total amount of circulating calcium, is biologically active; the remaining percentage is bound to plasma proteins (40%, of which albumin accounts for 90%, and globulins for 10%), or is in complex with anions (10%) such as citrate, lactate, bicarbonate, phosphate. Hypo- and hypercalcemia are common conditions treated by physicians of various specialities. Primary hyperparathyroidism and malignant tumors are the most common causes of hypercalcemia, whereas hypocalcemia is most often caused by hypoparathyroidism, malabsorption, vitamin D deficiency or chronic kidney disease. The interpretation of blood calcium concentration results affects the correct diagnosis, the need for further examination, and the choice of treatment. Concentration of ionized calcium is considered a more accurate indicator of the true status of calcemia compared to the concentration of total calcium, but its measurement is difficult due to strict preanalytical and analytical requirements. In the mid-1970s, calcium adjustment equations appeared, which are widely used today. However, some studies have expressed doubts about the sufficient reliability and sensitivity of the corresponding adjustment formulas. The diagnostic accuracy of widely used correction formulas in some clinical situations is lower than the diagnostic accuracy of uncorrected total calcium. The review discusses the history of the formulas for correcting total calcium for albumin, the factors influencing the result of correction, as well as its suitability in various conditions.

## ВВЕДЕНИЕ

Кальций является самым распространенным минералом в организме человека [1]. Около 99% кальция депонируется в костной ткани в виде солей гидроксиапатита и только 1% находится во внутриклеточном и внеклеточном (кровь и интерстициальная жидкость) пространстве [2]. Биологически активным является ионизированный кальций, составляющий примерно 50% от общего количества циркулирующего кальция, остальная часть связана с белками плазмы (40%, при этом в 90% случаев это альбумин, в 10% — глобулины) или находится в комплексе с анионами (10%), такими как цитрат, лактат, бикарбонат, фосфат [1][2]. Гипо- и гиперкальциемия — частые состояния, с которыми сталкиваются врачи различных специальностей. Первичный гиперпаратиреоз (ПГПТ) и злокачественные новообразования являются наиболее распространенными причинами гиперкальциемии, тогда как гипокальциемия чаще всего обусловлена гипопаратиреозом, мальабсорбцией, дефицитом витамина D или хронической болезнью почек (ХБП) [2]. Интерпретация результатов определения уровня кальция в крови влияет на постановку правильного диагноза, необходимость назначения дальнейшего обследования, выбор лечения. В обзоре обсуждается история возникновения формул коррекции общего кальция на альбумин, факторы, влияющие на результат коррекции, а также ее целесообразность при различных состояниях.

## ОПРЕДЕЛЕНИЕ КАЛЬЦИЯ В КРОВИ И ФАКТОРЫ, ВЛИЯЮЩИЕ НА РЕЗУЛЬТАТ

В сыворотке крови можно измерить концентрацию как общего, так и ионизированного («свободного») кальция. Совокупность фракций ионизированного кальция и кальция, связанного с анионами, обозначается как «способный к диффузии» или «ультрафильтрованный» кальций [3]. Исследование уровня общего кальция методами спектрофотометрии стало широкодоступным с 1970-х гг., когда появились автоматические биохимические анализаторы, позволившие внедрить его в рутинную практику [1]. Однако поскольку биологически активным является ионизированный кальций, считается, что именно его концентрация отражает истинное содержание кальция в крови, и его уровень является «золотым стандартом» для оценки кальциемии по сравнению с концентрацией общего кальция в крови [1][3]. Измерение ионизированного кальция затруднено ввиду строгих преаналитических и аналитических требований, что ограничивает его использование в широкой клинической практике [1][3], однако не все исследователи согласны с тем, что это должно быть препятствием к его определению [4–6]. Ионизированный кальций измеряется только методом потенциометрии с помощью ион-селективных электродов, и до сих пор по техническим причинам в крупные биохимические анализаторы опция прямого измерения ионизированного кальция не встроена, что также ограничивает доступность его определения [1].

В целом, в норме все три фракции кальция в крови (ионизированный, связанный с белками и находящийся в комплексе с анионами) находятся в равновесии, однако в ряде случаев (изменения в концентрации альбумина, изменения рН крови, жирные кислоты, связывающие альбумин, присутствие в сыворотке патологических белков, например, моноклональных иммуноглобулинов, наличие анионов, способных связывать кальций) равновесие между общим и ионизированным кальцием может быть нарушено [1]. Кроме того, на уровень общего кальция влияют изменение положения тела и длительное сдавление вены жгутом [7]. Основные причины несоответствия общего и ионизированного кальция представлены в таблице 1 (адаптировано из [8]).

**Table:** Таблица 1. Возможные причины несоответствия между измеренным общим кальцием и ионизированным кальцием (адаптировано из [8])

Общий кальций	Ионизированный кальций	Причины	Пояснение
↓	N	Гипоальбуминемия (например, печеночная недостаточность, нефротический синдром)	Снижение фракции кальция, связанного с альбумином
↑	N	Гиперальбуминемия (например, длительное наложение жгута, диета с высоким содержанием белка, длительное и выраженное обезвоживание)	Увеличение фракции кальция, связанного с альбумином
Множественная миелома	В редких случаях моноклональные глобулины связывают кальций, вызывая повышение общего содержания кальция В большинстве случаев при множественной миеломе преобладает истинная гиперкальциемия
N	↓	Хроническая болезнь почек	Сопутствующий метаболический ацидоз и почечная недостаточность
Острый респираторный алкалоз	Ионизированный кальций связывается с альбумином вместо ионов водорода
Хронический респираторный алкалоз	Почечная резистентность к паратиреоидному гормону вызывает гиперкальциурию
↑ / N	↓	Хелатирование цитратом (например, пациенты на заместительной почечной терапии)	Сопутствующее хелатирование ионизированного кальция и образование комплексов цитрата кальция
↑	↑↑	Первичный гиперпаратиреоз	Снижение связывания ионизированного кальция с альбумином сопровождается повышением отношения ионизированного кальция к общему кальцию

## ИСТОРИЯ СОЗДАНИЯ КОРРЕКТИРУЮЩИХ ФОРМУЛ

Еще в 1935 г. МакЛин и Гастингс опубликовали номограмму, позволявшую вычислить концентрацию ионизированного кальция исходя из концентрации общего кальция и белка (табл. 2) [9]. В последующем от использования номограмм отказались, и их место заняли формулы по расчету «скорректированного» кальция. При этом в качестве переменных в этих формулах использовались уровни белка; альбумина; альбумина и глобулинов; альбумина, глобулинов и рН (табл. 2). В целом, использование формул по коррекции уровня общего кальция преследует одну цель — более точное отражение уровня ионизированного кальция в крови для диагностики гипо-, нормо- или гиперкальциемии (т.е. заменить исследование ионизированного кальция неким суррогатным маркером, более простым в измерении). В 1971 г. Оррелл опубликовал статью «Альбумин как помощник в интерпретации уровня кальция в крови», где указал, что, по наблюдениям в их лаборатории, корреляция между общим кальцием и альбумином в два раза лучше, чем корреляция между общим кальцием и общим белком [10]. Автор исследовал 954 образца сыворотки крови как госпитализированных, так и амбулаторных пациентов на уровень общего кальция, белка и альбумина независимо от возраста и диагноза. При этом образцы с концентрацией кальция менее 7 мг/дл (около 1,75 ммоль/л) и более 11 мг/дл (около 2,75 ммоль/л) были исключены из исследования. Автор рассчитал коэффициент регрессии кальция на альбумин и вывел формулу коррекции кальция на альбумин:

Са (скорр) (мг/100 мл) = общий кальций (мг/100 мл) – 0,71 х [ альбумин (г/100 мл) – 3,4]

**Table:** Таблица 2. Формулы для коррекции общего кальция, опубликованные в 1930–1970-е гг. (адаптировано из [24]) Примечания: Cac — скорректированный кальций; Cat — общий кальций (г/дл); А — альбумин (г/дл); Р — общий белок (г/дл); G — глобулин = Р-А

Автор (ссылка в работе Ladenson)	Алгоритм
Коррекция, основанная на уровне общего белка
Dent (10)	Cac = Cat – 0,675 x (P – 7,2)
Parfitt (11)	Cac = Cat /(0,6 + 0,0541 x P)
Parfitt (12)	Cac = Cat /(0,55 + 0,062 x P)
Husdan (4, 6)	Cac = Cat /(0,6 + 0,0515 x P)
Christiansen (13)	Экстраполированный кальций Где α — фракция, связанная с белком. Если принять α за 40%, уравнение будет выглядеть так: Cae = Cat /(0,6 + 0,0533 x P)
Zeisler (14)	Ca++ = (6 x Cat – P/3)/(P + 6)
Kelly (15)	Са index = (Cat – 6)/(0,5 x P)
Коррекция, основанная на уровне альбумина
Orrell (16)	Cac = Cat – 0,707 x (A – 3,4)
Berry (7)	Cac = Cat – 0,91 x (A – 4,6)
Payne (8)	Cac = Cat – 0,989 x A + 4,0
Коррекция, основанная на уровне общего белка и альбумина
Pottgen & Davis (17)	
Коррекция, основанная на уровне альбумина, глобулина и рН
Moore (2)	Cac = Cat – [Ca – P], где [Ca – P]
Алгоритм для предсказания уровня ионизированного кальция
McLean&Hastings (1)	

При этом автор отметил, что точность измерения скорректированного кальция не так хороша, как общего кальция, поскольку лабораторная погрешность при измерении как общего кальция, так и альбумина вносят вклад в итоговый результат, однако не считал это критичным [10].

Однако основоположником формулы по расчету кальция, скорректированного на альбумин, считается британский химик Брайан Р. Пейн. В 1973 г. Пейн и соавт. опубликовали статью, поднимающую вопрос об интерпретации уровня общего кальция в крови у пациентов с гипергаммаглобулинемией [11]. В исследование авторы включили 200 образцов крови, присланных в лабораторию для определения печеночных проб (то есть у пациентов не предполагалось наличие нарушений обмена кальция, и при этом не брались образцы крови пациентов из отделений нефрологии), и измерили концентрацию общего кальция, белка и альбумина. Целью было создание формулы для скрининга пациентов с патологической концентрацией белка, чтобы иметь возможность учитывать эффект изменения связанного с белком кальция на измерение общего кальция. Авторы выявили, что уровень общего кальция хорошо коррелирует с уровнем альбумина (r=0,867) и хуже — с уровнем общего белка (r=0,682), и предложили следующую формулу для коррекции уровня общего кальция на альбумин:

Са (скорр) (мг/100 мл) = общий кальций (мг/100 мл) – альбумин (г/100 мл) + 4,0

или:

Са (скорр) (ммоль/л) = общий кальций (ммоль/л) – [ 0,025 х альбумин (г/л)] + 1,0

(в исходной статье [11] была допущена ошибка (был указан коэффициент 0,25, в последующем заменен на 0,025 [12]), а далее в статьях эта формула трансформировалась в

Са (скорр) (ммоль/л) = общий кальций (ммоль/л) + 0,025 х [ 40 — альбумин (г/л)] или же Са (скорр) (мг/дл) = общий кальций (мг/дл) + 0,8 x [ 4,0 — альбумин (г/дл)] — прим. авт.)

Авторы сделали вывод, что невозможно интерпретировать уровень кальция в крови, не зная уровень белка, и что поправка должна осуществляться с учетом уровня альбумина, а не общего белка [11].

Примечательно, что в этом же номере журнала от 1973 г. была опубликована статья Берри и соавт., в которой описывалось исследование в крови у 25 здоровых мужчин в возрасте 21–35 лет общего кальция, общего белка, альбумина, удельной плотности и ионизированного кальция до и после сдавления руки жгутом [7]. Авторы вывели свое уравнение коррекции кальция на альбумин, по которому нужно вычитать 0,09 мг/100 мл кальция на каждое увеличение на 0,1 г в уровне альбумина выше 46 г/100 мл, и добавлять, соответственно, при уровне альбумина меньше 46 г/100 мл (табл. 2). Также авторы обнаружили, что концентрация ионизированного кальция, в отличие от общего кальция, не изменяется при длительном сдавлении вены жгутом [7].

После публикации Пейна появились различные комментарии с критикой формулы [13—16], в частности, что приведенное уравнение не применимо в случаях гипо- и гиперкальциемии [14], на некоторые из которых авторы давали комментарии [17—19], в том числе указывали, что разработали уравнение как скрининговую процедуру для того, чтобы выбрать среди пациентов с отклоняющимися от нормы значениями альбумина тех, кому может потребоваться дальнейшее исследование гомеостаза кальция [19]. В последующем появились многочисленные исследования, ставящие под сомнение целесообразность использования этой формулы (см. ниже). Примечательно, что спустя почти 50 лет после публикации оригинальной работы, Пейн продолжал активное участие в обсуждении вопроса коррекции кальция на альбумин [20].

В 1977 г. в том же журнале, где была опубликована формула Пейна, вышла статья, в которой предлагалось, с учетом разброса поправочных коэффициентов в опубликованных работах (0,018, 0,019, 0,020, 0,021, 0,022, 0,023, 0,025), использование поправочного коэффициента 0,02, то есть увеличение концентрации общего кальция на 0,02 ммоль/л на каждый грамм снижения концентрации альбумина по сравнению с исходным значением 40 г/л (и наоборот): Ca (скорр) (ммоль/л) = общий кальций (ммоль/л) + 0,02 х [ 40 — сывороточный альбумин (г/л)] [3]. Автор статьи отмечал, что выбранное референсное значение альбумина должно быть в середине референсного интервала для используемого метода определения альбумина, и для большинства существующих (на тот момент) методов это значение равно 40. Кроме того, автор отмечал, что референсный интервал для «скорректированного» кальция каждая лаборатория должна определять самостоятельно [3]. За этой статьей также последовала критика, поскольку индивидуальный разброс поправочных коэффициентов может быть гораздо шире [21], и дальнейшая дискуссия [22][23]. Тем не менее именно эта формула используется наиболее часто в настоящее время под названием модифицированной формулы Пейна.

Ладенсон и соавт. в 1978 г. проанализировали все опубликованные на тот момент алгоритмы для коррекции уровня кальция (табл. 2), включая формулу Пейна, а также локально выведенный алгоритм на большой выборке как здоровых добровольцев, так и больных с различными патологиями (включая гиперкальциемию, злокачественные заболевания, ХБП и пр.), и убедились, что скорректированный кальций ни по одному из алгоритмов не предсказывал уровень ионизированного кальция правильнее, чем общий кальций [24].

В 1979 г. Пейн и соавт. проанализировали результаты 1673 образцов крови, отправленных на анализ по поводу костной или печеночной патологии, при этом исключались образцы из нефрологического отделения [25]. Для расчета альбумин-скорректированного кальция авторы использовали свою же формулу [11], при этом анализу подверглись две группы: 115 человек, у которых общий кальций был значимо ниже (<2,0 ммоль/л) или значимо выше (>2,75 ммоль/л) референсного интервала, и 74 пациента, имевшие «нормальный» уровень общего кальция, который переставал быть «нормальным» после коррекции. В первой группе после коррекции кальций отклонялся от нормы только у 24 пациентов (21%). Во второй группе ни у кого кальций не был ниже 2 ммоль/л, но был выше 2,75 ммоль/л у 6 больных. Из них у двух, по выводам авторов, альбумин был ошибочно занижен лабораторией, у остальных имелись злокачественные заболевания, тяжелое поражение печени или первичный гиперпаратиреоз. Примечательно, что в качестве контроля авторы не использовали ионизированный кальций, поэтому полученные выводы о достоверности результатов сомнительны. В этой же работе авторы обращают внимание на то, что корректировочная формула разработана для определения альбумина методом бромкрезолового зеленого (см. ниже) [25]. В последующем Барт и соавт. (в соавторстве с Пейном) подтвердили наблюдения других авторов (Эшби и соавт., см. ниже), что соотношение кальция и альбумина становятся нелинейными при низком уровне альбумина, что уравнения регрессии отличаются при использовании одинаковых приборов и методов исследования в разных лабораториях, и пришли к выводу, что каждая лаборатория должна рассчитывать свое уравнение регрессии кальция на альбумин из своих баз данных и проверять их достоверность время от времени [26]. В дальнейшем были предложены другие формулы для расчета альбумин-скорректированного кальция, преимущественно для больных с ХБП (табл. 3), однако именно модифицированная формула Пейна до сих пор применяется чаще всего.

**Table:** Таблица 3. Формулы для коррекции общего кальция, опубликованные после работы Пейна

Формула	Число пациентов	Авторы, год	Ссылка
Са (скорр) (ммоль/л) = общий кальций (ммоль/л) + 0,018 х (35 – альбумин (г/л))	50 пациентов на программном гемодиализе	Clase C.M. et al., 2000	[44]
Ионизированный кальций (ммоль/л) = 0,592 – 0,00449 х белок (г/л) + 0,410 х общий кальций (ммоль/л)	294 пациента 80 лет и старше с уровнем альбумина менее 35 г/л	Pfitzenmeyer P. et al., 2007	[82]
Са (скорр) (ммоль/л) = общий кальций (ммоль/л) + 0,01 х (30 – альбумин (г/л))	Разработка формулы: 60 пациентов на программном гемодиализе Подтверждение формулы: 237 пациентов на программном гемодиализе	Jain A. et al., 2008	[67]
Са (скорр) (ммоль/л) = общий кальций (ммоль/л) + 0,012 х (39,9 – альбумин (г/л))	Разработка формулы: 4613 амбулаторных пациентов Подтверждение формулы: дополнительные 1538 человек	James M.T. et al., 2008	[42]
Са (скорр) (ммоль/л) = общий кальций (ммоль/л) + 0,015 х (40 – альбумин (г/л)) + 0,07 х (1,5 – фосфор (ммоль/л))	84 пациента на программном гемодиализе	Ferrari P. et al., 2009	[75]
Ca (скорр) (ммоль/л) = общий кальций (ммоль/л) + 0,03 х (37,33 – альбумин (г/л))	Разработка формулы: 450 здоровых лиц 35–65 лет Подтверждение формулы: 125 здоровых лиц 35–65 лет	Pawade Y.R. et al., 2012	[64]
Са (скорр) (мг/дл) = общий кальций (мг/дл) + 0,25 х (4 – альбумин (г/дл)) + 4 х (7,4 – pH) + 0,1 х (6 – фосфор (мг/дл)) + 0,22	1922 пациента с хронической болезнью почек 3–5 стадии + 341 пациент с хронической болезнью почек на программном гемодиализе	Kaku Y. et al., 2015	[76]
Са (скорр) (мг/дл) = общий кальций (мг/дл) + 0,25 х (4 – альбумин (г/дл)) + 0,1 х (6 – фосфор (мг/дл)) + 0,05 х (24 – HCO3- (мг/дл)) + 0,35
Са (скорр) (ммоль/л) = 1,35 х общий кальций (ммоль/л) – 0,7 х альбумин (г/л) – 0,25	Разработка формулы: 242 пациента на программном гемодиализе Подтверждение формулы: 566 пациентов на программном гемодиализе	Obi Y. et al., 2016	[73]
Са (скорр) (ммоль/л) = 1,35 х общий кальций (ммоль/л) – 0,65 х альбумин (г/л) – 0,15 х фосфор (ммоль/л) – 0,25
Са (скорр) (ммоль/л) = 1,35 х общий кальций (ммоль/л) – 0,55 х альбумин (г/л) – 0,15 х фосфор (ммоль/л) – 0,005 х альбумин (г/л) х HCO3- (ммоль/л) + 0,4

Интересен вопрос референсного интервала для альбумин-скорректированного кальция. В целом общепринято, что он совпадает с референсным интервалом для общего кальция. Однако в 2020 г. Шини и соавт. опубликовали исследование, в котором рассчитали 99% референсный интервал для альбумин-скорректированного кальция [27]. Авторы проанализировали базу данных из 502 524 мужчин и женщин в возрасте от 40 до 69 лет, и отобрали группу из 178 377 человек без ХБП и дефицита витамина D. При расчете референсный интервал для общего кальция составил 2,15–2,6 ммоль/л. Альбумин-скорректированный кальций рассчитывался по выведенной авторами формуле:

Са (скорр) (ммоль/л) = общий кальций (ммоль/л) + 0,0177 х [ 45,2 — альбумин (г/л)].

Референсный интервал для альбумин-скорректированного кальция составил 2,19–2,56 ммоль/л, при этом при расчете отдельно для женщин в менопаузе верхняя граница референсного интервала составила 2,59 ммоль/л, для молодых женщин — 2,57 ммоль/л, а для всех мужчин — 2,55 ммоль/л [27].

## ЗАВИСИМОСТЬ ПОКАЗАТЕЛЯ АЛЬБУМИН-СКОРРЕКТИРОВАННОГО КАЛЬЦИЯ ОТ ФАКТОРОВ, СВЯЗАННЫХ С АЛЬБУМИНОМ

На молекуле альбумина находится около 30 мест связывания кальция [35], и этот кальциевый буфер сильно зависит от pН. Снижение рН приводит к повышению уровня ионизированного кальция, а повышение рН — к его снижению [1]. Концепция поправки кальция на альбумин предполагает постоянный коэффициент связывания кальция с альбумином (так, модифицированная формула Пейна предполагает добавление или вычитание 0,02 ммоль/л кальция на каждый г/л альбумина, на который альбумин в крови меньше или больше 40 г/л). Однако в работе Бесараб и соавт. было показано in vitro, что константа связывания значительно изменяется в зависимости от концентрации альбумина в диапазоне от 1 до 9 г/дл [28]. При постоянной концентрации альбумина концентрация кальция, связанного с альбумином, изменялась непосредственно с концентрацией ультрафильтруемого кальция. При постоянной концентрации ультрафильтруемого кальция концентрация кальция, связанного с альбумином, была обратно пропорциональна концентрации альбумина при уровне альбумина более 3 г/дл. В результате авторы делают вывод, что связывание кальция с альбумином — сложный процесс, характеризующийся наличием множества мест связывания, чья аффинность и способность к связыванию различается, и что использование простых формул с фиксированной константой связывания кальция с альбумином может быть некорректно, особенно при гипоальбуминемии [28]. В последующем этими же авторами в исследовании in vivo было показано, что количество кальция, связанного с 1 г альбумина, обратно коррелирует с концентрацией альбумина: снижение с 2 до 1 мг кальция на грамм альбумина с увеличением концентрации альбумина с 1,7 до 3,1 г/дл [29]. Это исследование доказывает, что использование корректировочных формул с константой связывания кальция с альбумином у лиц с гипоальбуминемией приводит к ложному завышению у них уровня кальция, т.е. у лиц с низким уровнем ионизированного кальция ложно диагностируют нормокальциемию [29].

Сродство связывания кальция с альбумином также имеет значительные индивидуальные различия. Фактически индивидуальные поправочные коэффициенты (коэффициенты регрессии) составляют от 0,013 до 0,044 [30]. Факторы, влияющие на связывание кальция с альбумином, могут различаться у амбулаторных пациентов и при состояниях, требующих неотложного медицинского вмешательства. Пациенты, страдающие каким-либо заболеванием, могут иметь даже большие различия в связывающей способности кальция с альбумином, чем здоровые люди, что делает использование «среднего» поправочного коэффициента 0,02 еще более сомнительным [30]. Имеются ограниченные данные, что концентрация альбумина изменяется в зависимости от пола, возраста и между популяциями, однако не все исследования согласуются между собой. Например, в работе МакПерсон и соавт. продемонстрировано снижение уровня альбумина на 10% в возрастном диапазоне 18–65 лет у 1000 здоровых доноров крови. Значения сывороточного альбумина были ниже в среднем на 2 г/л у женщин до возраста менопаузы [31]. В другом исследовании, включившем 1 079 193 результата анализа сывороточного альбумина от 482 607 мужчин и 596 586 женщин, показано увеличение средней концентрации альбумина до максимальной примерно в возрасте 20 лет, а затем постепенное ее снижение, причем у женщин наблюдалось более быстрое уменьшение альбумина к возрасту 60 лет [32].

В 1986 г. Эшби и соавт. проанализировали базу данных из 687 000 образцов крови, при этом были исключены образцы с отклоняющимися от нормы уровнями мочевины, фосфора, глобулинов, щелочной фосфатазы и печеночных ферментов [33]. Коэффициенты регрессии кальция на альбумин изменялись значительно на различных приборах, даже если аналитические принципы используемых методов оставались одинаковыми. Кроме того, взаимосвязь общего кальция и альбумина отклонялась от линейной при уровне альбумина менее 30 г/л. Также авторы отметили, что при использовании большой базы данных можно вычислить референсный интервал для альбумин-скорректированного кальция вместо того, чтобы каждый раз рассчитывать скорректированный кальций [33]. В последующем Барт и соавт. подтвердили, что взаимосвязь кальция и альбумина отклоняется от линейной при низких показателя альбумина, что в различных лабораториях поправочные коэффициенты варьируют от 0,016 до 0,0232 [26].

Коррекция кальция на альбумин затруднена в связи с существованием различных наборов для определения концентрации альбумина [34][35]. Концентрация альбумина обычно измеряется фотометрическими методами, при этом в качестве красителя используется либо бромкрезоловый пурпурный (БКП), либо бромкрезоловый зеленый (БКЗ) (следует отметить, что в работе Пейна для определения альбумина использовался БКЗ). При этом «золотым стандартом» измерения уровня альбумина являются иммунохимические методы (иммунотурбидиметрия и иммунонефелометрия), однако они более трудозатратные и дорогие [34]. Примечательно, что, особенно у больных на гемодиализе, показатели альбумина, измеренные методами БКП и БКЗ, отличаются от измеренных иммунохимическими методами: метод БКП занижает показатели альбумина, а БКЗ завышает, особенно при гипоальбуминемии (в связи с тем, что связывает в небольшом количестве еще и глобулины) [36][37]. Таким образом, значение кальция, скорректированного на альбумин, зависит от набора, которым определяли уровень альбумина: при использовании БКП уровень скорректированного кальция может быть завышен, а при использовании БКЗ — занижен. В исследовании Лабриола и соавт. изучали зависимость уровня альбумин-скорректированного кальция от метода измерения альбумина у пациентов на гемодиализе [34]. В исследование включили 89 больных, уровень альбумина составил 3,78±0,24 г/дл методом БКЗ и 3,12±0,27 г/дл методом БКП (р<0,0001). Основываясь на уровне альбумина, измеренного методом БКЗ, было 12 случаев «гипокальциемии» (8,6 мг/дл), 3 случая «гиперкальциемии» (>10 мг/дл) и 74 случая «нормокальциемии». Основываясь на уровне альбумина, измеренного методом БКП, у одного пациента была «гипокальциемия», у 21 — «гиперкальциемия» и у 67 — «нормокальциемия». Таким образом, в зависимости от метода измерения альбумина (БКП или БКЗ) — разногласия в результатах в одной и той же когорте больных получены в 29 случаях (32,6%) [34]. Авторы делают вывод, что при измерении уровня альбумина лаборатории должны указывать метод, каким он был измерен [34]. В исследовании де Рой ван Зюйдевейн и соавт. измеряли уровень альбумина методом БКП у 331 пациента и методом БКЗ у 175 пациентов на гемодиализе [35]. В группе БКП уровень альбумина был ниже (34,5±4,2 против 40,3±3,1 г/л, p<0,0005). Уровень общего кальция не отличался между группами (2,32±0,18 ммоль/л и 2,34±0,17 ммоль/л в группах БКП и БКЗ соответственно (р=0,26)), тогда как после коррекции на альбумин кальция стал значительно выше в группе БКП (2,45±0,18 против 2,33±0,18 ммоль/л, p<0,0005) [35]. Авторы приходят к выводу, что не следует корректировать уровень кальция на альбумин у больных на гемодиализе при выборе фосфат-биндеров [35]. Схожие данные по различиям в концентрации альбумина, исследованной методами БКЗ и БКП, и, соответственно, по альбумин-скорректированному кальцию у пациентов на гемодиализе также получены в исследовании Като и соавт. [38]. В исследовании Карневале и соавт. исследовали уровень общего кальция, общего белка и альбумина, измеренного двумя методами — колориметрическим и электрофоретическим — у 170 госпитализированных больных [39]. При колориметрическом методе уровень альбумина был значимо ниже, чем при электрофоретическом, и, соответственно, уровень альбумин-скорректированного кальция по формуле Пейна был значимо ниже во втором случае, что в конечном итоге влияло на частоту выявленных случаев гиперкальциемии [39]. В двух исследованиях Джэссэм и соавт. как показатели альбумина, так и показатели кальция значительно изменялись в зависимости от используемого оборудования и реагентов, что может сильно влиять на конечный результат альбумин-скорректированного кальция [40][41].

С учетом того, что в корректировочных формулах, разработанных в 1970-х гг. (табл. 2), для определения альбумина использовался БКЗ, в 2008 г. Джеймс и соавт. разработали формулу для БКП (табл. 3) [42].

## В КАКИХ КЛИНИЧЕСКИХ РЕКОМЕНДАЦИЯХ РЕКОМЕНДУЮТ КОРРЕКТИРОВАТЬ ОБЩИЙ КАЛЬЦИЙ НА АЛЬБУМИН

## Минеральные и костные нарушения при ХБП

Как гипокальциемия, так и гиперкальциемия — предикторы смертности у больных на гемодиализе, от их наличия зависит выбор лечения (назначение активных метаболитов витамина D, назначение кальций-содержащих или не содержащих фосфат-биндеров) [43]. В рекомендациях K/DOQI от 2003 г. указано (рекомендация 6, стр. S77): «В большинстве случаев воспроизводимость измерений ионизированного кальция хуже, чем общего кальция; методика является времязатратной и более дорогой по сравнению с измерением общего кальция. По этой причине, а также потому, что ионизированный кальций не измеряется в рутинной практике, Рекомендации будут основаны на уровне общего кальция в крови. Последний отражает концентрацию ионизированного кальция, если уровень белка в крови находится в норме. Если уровень альбумина низкий, необходимо проводить коррекцию измеренного уровня общего кальция. Несколько формул было разработано для коррекции общего кальция на отклоняющийся от нормы альбумин как у здоровых лиц, так и у пациентов с ХБП, но все они имеют ограничения» [43]. В рекомендациях приведены две формулы для коррекции общего кальция на альбумин. Одна из них взята из исследования Клейз и соавт. [44]:

Са (скорр) (мг/дл) = общий кальций (мг/дл) + 0,0704 х [ 34 — альбумин (г/л)],

где альбумин измерялся на автоматическом анализаторе методом БКЗ, общий кальций методом фотометрии с арсеназо III, а ионизированный кальций — ион-селективными электродами.

При этом авторы рекомендаций отмечают, что использование других методов для измерения как альбумина, так и кальция, может приводить к другим корреляциям, нежели в приведенном уравнении. И для рутинной клинической интерпретации уровня кальция в крови у пациентов с ХБП авторы рекомендаций предлагают использовать модифицированную формулу Пейна. В указанной главе формула приведена в следующем контексте: «Дегидратация или гемоконцентрация при венепункции могут повысить уровень альбумина и ложно завысить уровень общего кальция. Даже при нормальном уровне альбумина, изменения рН крови нарушают константу равновесия комплекса кальций-альбумин, при этом ацидоз уменьшает связывание, а алкалоз уcиливает. Соответственно, глобальные сдвиги в концентрации белка и рН требуют прямого измерения ионизированного кальция для определения физиологического уровня кальция» [43].

В клинических рекомендациях KDIGO, опубликованных в 2006 г., указано, что судить об уровне кальция у пациентов с ХБП необходимо либо по уровню ионизированного кальция, либо по альбумин-скорректированному кальцию [45]. При этом авторы отмечают, что измерение ионизированного кальция является методом выбора. Однако сложности, связанные с обработкой образца, и стоимость исследования могут препятствовать его рутинному измерению. Уровень общего кальция должен быть «скорректирован» на уровень альбумина при низком уровне кальция, однако не существует стандартизации используемых формул для определения «скорректированного кальция» [45]. При формулировке этих утверждений авторы ссылаются на рекомендации K/DOQI. Среди вопросов, необходимых для изучения в дальнейшем, приводят следующий: обеспечивают ли существующие формулы, используемые для «корректировки» общего кальция в крови, и основанные на уровне альбумина в крови, более точное отображение уровня кальция в крови по сравнению с некорректированным уровнем общего кальция [45].

В последующем в рекомендациях KDIGO, опубликованных в 2009 г., было указано (глава 3.1, раздел 3.1.6, стр. S27): «При наличии гипоальбуминемии ионизированный кальций повышается относительно общего кальция и, таким образом, показатель общего кальция может недооценить уровень биологически активного (ионизированного) кальция [46]. Формула «скорректированного кальция» рутинно используется во многих лабораториях у пациентов на гемодиализе и в большинстве клинических исследований. К сожалению, последние данные показывают, что она не дает преимуществ по сравнению с уровнем общего кальция и менее специфична по сравнению с исследованием ионизированного кальция. Набор, используемый для измерения альбумина, может повлиять на оценку скорректированного кальция. К тому же измерение ионизированного кальция не доступно в рутинной практике и может требовать дополнительных затрат. В настоящее время большинство баз данных уже используют формулу для коррекции кальция и нет данных, показывающих разницу в лечебном подходе или клинических исходах при использовании скорректированного против общего или ионизированного кальция. Рабочая группа не рекомендует избегать коррекции кальция на альбумин. Кроме того, использование ионизированного кальция в настоящее время не считается практичным и экономически эффективным» [46]. В последних опубликованных рекомендациях KDIGO от 2017 г. не указано, какой именно кальций необходимо измерять у больных с ХБП [47].

## Первичный гиперпаратиреоз

В международных рекомендациях от 2002 г. указано, что концентрация общего кальция должна быть скорректирована на концентрацию альбумина по модифицированной формуле Пейна. Панель экспертов не рекомендовала использовать ионизированный кальций, поскольку у большинства клиницистов нет возможности его измерить [48]. В рекомендациях от 2008 г. коррекция кальция на альбумин не упоминается [49]. В рекомендациях от 2013 г. указано, что концентрация общего кальция корректируется, чтобы отразить любые отклонения в уровне альбумина. Рекомендуется использовать модифицированную формулу Пейна. Уровень ионизированного кальция может быть измерен, однако у большинства центров нет возможности для оценки ионизированного кальция. Таким образом, рекомендуется использовать скорректированную концентрацию общего кальция [50]. В рекомендациях от 2023 г. указано следующее: «Рекомендуется корректировать общий кальций на уровень альбумина при диагностике первичного гиперпаратиреоза. Коррекцию необходимо проводить при уровне альбумина менее 4 г/дл. Необходимость коррекции при уровне альбумина более 4 г/дл остается спорной. Если доступен анализатор для определения ионизированного кальция, спорные вопросы, связанные с уровнем альбумина, могут быть разрешены. Исследование уровня ионизированного кальция тем более актуально, когда уровень общего кальция в норме, а уровень паратгормона (ПТГ) повышен» [51]. В российских рекомендациях по первичному гиперпаратиреозу также рекомендуется корректировать уровень общего кальция на альбумин [52].

Примечательно, что во всех вышеперечисленных редакциях рекомендаций по ПГПТ как одно из показаний для хирургического лечения асимптомного ПГПТ выбран уровень кальция выше 0,25 ммоль/л (1 мг/дл) от верхней границы нормы, а уровень ионизированного кальция не рассматривается как показание к операции. При этом существует как минимум одно исследование, показывающее, что именно уровень ионизированного, а не общего кальция лучше коррелирует с тяжестью заболевания [53]. Кроме того, неясно, соответствует ли верхняя граница референсного интервала для альбумин-скорректированного кальция той же границе референсного интервала для общего кальция, указываемого лабораторией, а от этого зависит показание к оперативному вмешательству.

## Гипопаратиреоз

В международных рекомендациях от 2016 г. указано, что для диагностики гипопаратиреоза авторы рекомендуют использовать ионизированный или альбумин-скорректированный кальций, при этом для коррекции используют ту же модифицированную формулу Пейна. Авторы упоминают все те же недостатки в измерении ионизированного кальция и рекомендуют корректировать общий кальций на альбумин [54]. В рекомендациях от 2022 г. указано, что для диагностики гипопаратиреоза, а также для мониторинга эффективности терапии рекомендуется использовать ионизированный кальций или альбумин-скорректированный кальций, при этом не указано, по какой формуле необходимо корректировать кальций [55]. В российских рекомендациях по гипопаратиреозу рекомендуется измерять альбумин-скорректированный кальций [56].

## Другие рекомендации

Поскольку у большинства пациентов с множественной миеломой наблюдаются как гиперкальциемия, так и гипоальбуминемия, Альянс National Comprehensive Cancer Network (NCCN) рекомендует использовать определение общего кальция в сыворотке крови с поправкой на концентрацию альбумина в данной популяции больных [57]. В европейских рекомендациях по купированию гиперкальциемии [58] и гипокальциемии [59] при острых состояниях также рекомендуется корректировать общий кальций на альбумин.

## ЦЕЛЕСООБРАЗНОСТЬ КОРРЕКЦИИ ОБЩЕГО КАЛЬЦИЯ НА АЛЬБУМИН ПРИ РАЗЛИЧНЫХ СОСТОЯНИЯХ

В литературе опубликовано достаточно большое количество исследований из разных стран, ставящих под сомнение целесообразность коррекции общего кальция в крови на альбумин. Все исследования в целом построены по одному принципу. В качестве «золотого стандарта» брался уровень ионизированного кальция и исследовалось, насколько общий и альбумин-скорректированный кальций соответствовали уровню ионизированного кальция. При этом преобладал ретроспективный дизайн исследований, когорты исследуемых отличались по наличию различных заболеваний и по количеству.

## Неоднородные когорты пациентов и здоровых лиц

Во Франции Паран и соавт. ретроспективно проанализировали результаты 74 пациентов, у которых исследовались кальций общий, кальций ионизированный, скорректированный на рН 7,4, альбумин, и проводился расчет альбумин-скорректированного кальция по двум модификациям формулы Пейна (0,02/40 и 0,025/40) [60]. В результате обе формулы занижали уровень кальция при уровне альбумина более 40 г/л, достигая разницы в 0,2 ммоль/л при уровне альбумина более 44 г/л. Использование этих формул также «маскировало» гиперкальциемию [60]. В Канаде Стин и соавт. ретроспективно проанализировали уровни общего кальция, альбумина и ионизированного кальция в 678 пробах от 420 госпитализированных пациентов независимо от диагноза [61]. Диагностическая точность альбумин-скорректированного кальция по формуле Пейна и нескорректированного общего кальция оценивалась в когорте госпитализированных пациентов с острым, но не критическим состоянием. Согласно полученным результатам, чувствительность и специфичность общего кальция в выявлении гипо- и гиперкальциемии составили 61% и 92%, 69% и 95% соответственно, тогда как коррекция общего кальция на альбумин по формуле Пейна демонстрировала чувствительность и специфичность в отношении выявления гипо- и гиперкальциемии — 95% и 76%, 20% и 99% соответственно. В то время как нескорректированный кальций неверно классифицировал статус кальция в 24% случаев, применение поправочной формулы на альбумин завышало значения кальция у 56% пациентов. При расчете альбумин-скорректированного кальция различные коэффициенты корреляции были выше для общего и ионизированного кальция по сравнению с альбумин-скорректированным и ионизированным кальцием. Среди ограничений своей работы авторы отмечают то, что среди госпитализированных (тяжело больных) чаще преобладает гипокальциемия, поэтому выборка в данном исследовании была смещена в сторону гипокальциемии, и полученные выводы могут не распространяться на пациентов с гиперкальциемией. Кроме того, ретроспективный дизайн работы не позволял проверить правильность забора и транспортировки образцов крови [61]. В Швеции Ридфельт и соавт. ретроспективно проанализировали результаты проб 20 003 пациентов, при этом для расчета альбумин-скорректированного кальция авторы использовали разработанную в лаборатории формулу (табл. 3) и семь других различных формул, опубликованных в литературе [62]. Авторы делают вывод, что уровень общего кальция лучше коррелирует с ионизированным кальцием (внутриклассовый коэффициент корреляции (interclass correlation coefficient, ICC) 0,85), чем альбумин-скорректированный кальций, рассчитанный по всем формулам (ICC варьировал в диапазоне от 0,45 до 0,81). По уровню общего кальция пациенты были верно классифицированы как имеющие гипер-, нормо- или гипокальциемию в 82% случаев, а результат по альбумин-скорректированному кальцию не стал лучше. Только в группе пациентов с гипоальбуминемией (альбумин менее 30 г/л) локальная формула классифицировала пациентов немного корректнее. Авторы делают вывод, что коррекция общего кальция на альбумин не добавляет значимой информации, и такой практики следует избегать. Следует отдавать предпочтение измерению ионизированного кальция, когда получены отклонения в уровне общего кальция, а также у пациентов с гипоальбуминемией [62]. В Австралии Смит и соавт. ретроспективно проанализировали 13 604 результата анализов от 5553 госпитализированных пациентов, а также проспективно — 1113 образцов от 450 пациентов [63]. В этой работе также было показано, что альбумин-скорректированный кальций хуже коррелировал с ионизированным кальцием и завышал уровень кальция у пациентов с гипоальбуминемией (уровень альбумина менее 3 г/дл) и у больных с почечной недостаточностью [63]. В ретроспективном исследовании во Франции Алан-Жела и соавт. среди 210 пациентов, госпитализированных в общетерапевтическое отделение, по результатам определения концентрации ионизированного кальция у 46 (22%) выявлена гипокальциемия, у 124 (59%) — нормокальциемия и у 40 (19%) — гиперкальциемия [64]. Концентрация общего кальция имела хорошую специфичность 94% (95% доверительный интервал (ДИ) 89–97%), но низкую чувствительность 52% (95% ДИ 37–67%) для диагностики гипокальциемии. Снижение уровня общего кальция указывало на гипокальциемию с хорошим уровнем достоверности (отношение правдоподобия положительного результата (positive likelihood ratio, PLR) 8,6 (95% ДИ 4,4–17)), однако нормальная концентрация общего кальция не исключала гипокальциемию (отношение правдоподобия отрицательного результата (negative likelihood ratio, NLR) 0,51 (95% ДИ 0,38–0,69). Поправка на концентрацию альбумина с использованием формулы Пейна значительно улучшала специфичность (p=0,008), но снижала чувствительность (p=0,001). Для диагностики гиперкальциемии общий кальций имел хорошую чувствительность 93% (95% ДИ 80–98%) и специфичность 89% (95% ДИ 83–93%). Высокая концентрация общего кальция указывала на гиперкальциемию (PLR 8,3 (95% ДИ 5,4–13)), в то время как нормальный уровень общего кальция в сыворотке крови исключал гиперкальциемию (NLR 0,08 (95% ДИ 0,03–0,25)) с хорошим уровнем достоверности. Однако корректировка общего кальция по концентрации альбумина не увеличивала, а иногда, наоборот, снижала точность диагностики (p<0,001). Таким образом, нескорректированный общий кальций столь же чувствителен, как и общий кальций с поправкой на альбумин, для выявления гиперкальциемии, однако измерение общего кальция, с коррекцией по уровню альбумина или без таковой, не во всех случаях позволяет диагностировать гипокальциемию. Приведенное исследование свидетельствует о том, что корректирующие формулы с использованием концентрации сывороточного альбумина и общего белка не повышают точность отражения истинного уровня кальция для диагностики гипокальциемии и даже ухудшают точность выявления гиперкальциемии [64].

Один из недостатков вышеперечисленных исследований — их ретроспективный дизайн. Однако схожие данные были получены и в проспективном исследовании Мир и соавт. [65]. В 254 образцах крови, присланных в лабораторию местного госпиталя (без уточнения диагнозов), измеряли уровень общего кальция, белка, альбумина, а также ионизированного кальция, при этом обязательным условием было нормальное значение рН в образце. Все образцы были разделены на три подгруппы: гипоальбуминемия (n=145), нормоальбуминемия (n=98) и гиперальбуминемия (n=11), и в каждой из подгрупп авторы рассчитывали скорректированный кальций по формулам Берри, Оррелла и Пейна, и рассчитывали ионизированный кальций как 50% от полученного результата. В итоге измеренный ионизированный кальций значимо отличался от расчетного по всем трем формулам ионизированного кальция в группе гипоальбуминемии (n=145), а также суммарно по всем образцам (n=254). Схожие результаты по измеренному и расчетному кальцию были получены только для расчета по формуле Оррелла в группе нормоальбуминемии и по формуле Берри в группе гиперальбуминемии. Авторы делают вывод, что лучше измерять ионизированный кальций, а при использовании формул необходимо помнить о всех присущих им ограничениях [65]. В проспективном исследовании Паваде и соавт. исследовали образцы 575 здоровых лиц 35–65 лет на общий кальций, ионизированный кальций и альбумин [66]. Авторы вывели собственное уравнение линейной регрессии для коррекции кальция на альбумин (табл. 3) и сравнивали полученный результат с результатом расчета по формуле Пейна. В результате полученные значения по двум разным формулам значимо отличались друг от друга, из чего авторы делают вывод, что каждая лаборатория должна рассчитывать собственные уравнения регрессии, и, кроме того, подобные уравнения, полученные на группах амбулаторных больных, не применимы к госпитализированным больным или к больным в тяжелом состоянии [66]. В проспективном многоцентровом исследовании Тод и соавт. с участием 1213 пациентов с подозрением на различные отклонения в обмене кальция диагностическое несоответствие между общим кальцием и ионизированным кальцием сыворотки крови обнаружено у 31% больных [67]. При расчете общего кальция с поправкой на альбумин и сопоставлении с уровнем ионизированного кальция у тех же пациентов выявленное несоответствие уменьшилось до 17,9%, при этом диагностическое несоответствие, которое объясняется аналитической неточностью, составило всего 6,7%. Можно предположить, что некоторым пациентам с повышенным уровнем общего кальция или кальция, скорректированного на альбумин, как следствие, будут проведены обследования, несмотря на нормальные значения ионизированного кальция, тогда как у других пациентов, вероятно, не будет дальнейшего обследования, несмотря на наличие гиперкальциемии, определяемой по уровню ионизированного кальция [67].

## Минеральные и костные нарушения при ХБП

Формула Пейна не была разработана для больных с нарушением функции почек, более того, при ее разработке такие больные специально не включались в исследование [11]. Тем не менее именно эта формула рекомендуется для расчета альбумин-скорректированного кальция у больных с ХБП [43]. В связи с этим проводились различные исследования об эффективности ее применения у больных с ХБП, а также попытки разработки других корректировочных формул. В исследовании Клейз и соавт. измеряли уровень ионизированного кальция, общего кальция, альбумина, общего белка и рН у 50 пациентов на гемодиализе в стабильном состоянии, и далее рассчитывали ICC между ионизированным кальцием и общим кальцием [44], а также кальцием, скорректированным по формулам Оррелла [10], Берри [7], Пейна [11], Маршалла и Ходжкинсона [68]. В результате ICC был выше для формул Оррелла и Маршала и Ходжкинсона, а также для общего кальция, но не для кальция, рассчитанного по формуле Пейна. Также авторы предлагают собственную разработанную формулу (табл. 3), которая очень близка к формуле Оррелла. Авторы делают вывод, что у пациентов на гемодиализе не следует проводить коррекцию общего кальция на альбумин, а в сомнительных случаях следует измерять ионизированный кальций [44]. В исследовании Джейн и соавт. на выборке из 60 пациентов на гемодиализе в стабильном состоянии и в дальнейшем при валидации на большей выборке из 237 пациентов на гемодиализе была разработана новая формула для расчета альбумин-скорректированного кальция (табл. 3), однако по показателю ICC эта формула значимо не превосходила формулы Оррелла, Клейза, а также показатель общего кальция [69]. В исследовании Горанссон и соавт. измеряли общий кальций, ионизированный кальций и альбумин-скорректированный кальций у 34 пациентов на гемодиализе [70]. При расчете альбумин-скорректированного кальция один пациент (3%) был отнесен в группу гипокальциемии, а 10 пациентов (26%) — в группу гиперкальциемии, тогда как по уровню ионизированного кальция пять пациентов (15%) и три пациента (9%) были отнесены в группы гипо- и гиперкальциемии соответственно. Таким образом, семь пациентов (21%) были неправильно классифицированы как имевшие гиперкальциемию по альбумин-скорректированному кальцию, и только 19 пациентов (56%) были классифицированы как имевшие нормокальциемию по обоим измерениям. Авторы приходят к выводу, что коррекция кальция на альбумин приводит к недооценке гипокальциемии и увеличению частоты гиперкальциемии в этой когорте больных [70]. В исследование Гаучи и соавт. был включен 691 пациент с ХБП 3–5 стадии с целью изучения возможности общего кальция и альбумин-скорректированного кальция прогнозировать низкие, нормальные и высокие концентрации ионизированного кальция, и авторы пришли к выводу, что альбумин-скорректированный кальций не превосходит общий кальций в диагностике отклонений значений ионизированного кальция, и что общий кальций имеет низкую чувствительность в диагностике как гипо-, так и гиперкальциемии: только около трети пациентов с гипокальциемией и одна пятая пациентов с гиперкальциемией были правильно классифицированы на основании уровня общего кальция [71]. Кроме того, низкий уровень tСО2 у пациентов с ХБП 3–5 (метаболический ацидоз) является независимым фактором риска занижения уровня кальция, однако этот показатель не включен в корретировочные формулы. Авторы рекомендуют измерять ионизированный кальций и рН у пациентов с ХБП 3–5 и низким tCO2 и/или альбумином в крови [71]. В обзоре Мортон и соавт., посвященном коррекции кальция на альбумин у больных на гемодиализе, опубликованном в 2010 г., авторы приходят к выводу о нецелесообразности коррекции кальция на альбумин у таких больных [72].

В 2015 г. в проспективном исследовании Джин и соавт. на выборке из 160 пациентов на гемодиализе было показано, что гипокальциемия по уровню ионизированного кальция наблюдалась в 40% случаев, а гиперкальциемия — в 1,8% [73]. При этом общий кальций верно предсказывал гипокальциемию в 48,4% случаях, а альбумин-скорректированный кальций по формуле Пейна — в 17,2%. Общий кальций не отражал гиперкальциемию, а альбумин-скорректированный кальций отражал в 2/3 случаев. Для выявления нормокальциемии общий кальций был точен в 92,4% случаев, а альбумин-скорректированный — в 88,1% [73]. В 2019 г. Пекар и соавт. ретроспективно проанализировали результаты 5055 образцов крови, где одновременно определялись общий кальций, альбумин, ионизированный кальций, рН и СКФ, при этом альбумин-скорректированный кальций далее рассчитывался по формулам Пейна, Клейза, Джейна и Ридфельта, и далее оценивалась согласованность результатов общего кальция и альбумин-скорректированного кальция, при этом «золотым стандартом» считался уровень ионизированного кальция [74]. Пациентов разделили на две группы: с альбумином менее 35 г/л (n=3049) и более 35 г/л (n=2006), и далее проводился анализ в подгруппах в зависимости от СКФ и рН. У пациентов с низким уровнем альбумина или отклонениями в значениях рН альбумин-скорректированный кальций по формуле Пейна завышал уровень кальция до 80% случаев. Общий кальций показывал хорошую согласованность с ионизированным кальцием в случаях гипоальбуминемии и не отличался от рассчитанного по формуле Пейна при нормальном уровне альбумина. Кальций, рассчитанный по формулам Клейза, Джейна и Ридфельта, лучше отражал уровень ионизированного кальция, но не превосходил общий кальций [74].

Несмотря на большое количество различных формул коррекции значения уровня кальция (табл. 2 и 3), сравнительно недавно оценку вновь разрабатываемым уравнениям стали давать не только по улучшению чувствительности и специфичности корректирующей формулы, но и с учетом предсказания определенных конечных исходов. Так, например, в исследовании Оби и соавт. с участием 874 пациентов на заместительной почечной терапии было показано, что больные с гиперкальциемией по уровню ионизированного кальция более 1,32 ммоль/л в 88% и 70% находились в нормальном диапазоне при оценке по скорректированному и нескорректированному общему кальцию [75]. В сравнении с пациентами с концентрацией ионизированного кальция в диапазоне от 1,16 до 1,24 ммоль/л больные с уровнем более 1,32 ммоль/л имели высокие риски летального исхода (отношение рисков (ОР) 1,77; 95% ДИ 1,13–2,75). Кроме того, пациенты со скрытой гиперкальциемией (повышенные уровни ионизированного кальция, но нормальные значения скорректированного и нескорректированного общего кальция) также имели высокие риски летального исхода — ОР 1,75 (95% ДИ 1,11–2,75) и ОР 1,80 (95% ДИ 1,11–2,90), соответственно. Авторы приходят к выводу, что большинство пациентов с терминальной стадией ХБП и повышенным содержанием ионизированного кальция ошибочно классифицируются как нормокальциемические при рутинном определении общего содержания кальция и имеют более высокий риск неблагоприятного исхода [75].

В более позднем ретроспективном исследовании Лайан и соавт., включившем 6549 пациентов, проводилось сравнение диагностической точности кальция, скорректированного на альбумин, рассчитанного по формуле

Са (скорр) (ммоль/л) = общий кальций (ммоль/л) + коэффициент × (40 — альбумин (г/л),

где коэффициент для каждой подгруппы пациентов с расчетной скоростью клубочковой фильтрации (рСКФ) выше или ниже 60 мл/мин/1,73 м², выводился с использованием модели множественной линейной регрессии), с нескорректированным общим кальцием и шестью другими формулами, доступными в литературе [76]. При множественной линейной регрессии у пациентов с рСКФ ≥60 мл/мин/1,73 м² и концентрацией альбумина <30 г/л скорректированный коэффициент регрессии был на 32,5% выше, чем у пациентов с рСКФ ≥60 мл/мин/1,73 м² и альбумином ≥30 г/л. Примечательно, что у пациентов с рСКФ ≥60 мл/мин/1,73 м² нескорректированный уровень кальция не уступал любым формулам с поправкой на альбумин в диагностике гипокальциемии, тогда как при рСКФ <60 мл/мин/1,73 м² нескорректированный кальций превзошел все формулы с поправкой на альбумин в диагностике гипокальциемии (p<0,001). Таким образом, диагностическая точность нескорректированного кальция в целом превосходит точность альбумин-скорректированного кальция у пациентов на разных стадиях ХБП. Однако при диагностике гиперкальциемии нескорректированный кальций оказался несколько хуже, чем некоторые формулы с поправкой на альбумин. Авторы также делают вывод, что уровня общего кальция достаточно для оценки кальциемии и использование корректировочных формул нецелесообразно, а если клиницист не доверяет уровню общего кальция, необходимо измерять уровень ионизированного кальция [76].

В 2009 г. Феррари и соавт. предложено включить в корректировочную формулу для пациентов на гемодиализе показатель фосфора в крови (табл. 3) [77], а позднее Каку и соавт. [78] соединили в одной формуле возможность поправки одновременно на уровень альбумина, фосфора и pH (табл. 3).

Кроме того, в исследовании Эвенпоэль и соавт. было показано, что измерение общего кальция «маскирует» гиперкальциемию у пациентов после трансплантации почки (у 58% — гиперкальциемия по ионизированному кальцию vs 13% по общему кальцию), и коррекция общего кальция на альбумин не улучшала диагностическую точность [79].

Таким образом, как общий, так и альбумин-скорректированный кальций, имеют низкую чувствительность (40–50%) но высокую специфичность (около 90%) для диагностики гипокальциемии и очень низкую чувствительность (20–30%) и высокую специфичность (90–100%) для диагностики гиперкальциемии у больных ХБП [64][71][73].

## Пожилые пациенты и больные в тяжелом состоянии

У пациентов в тяжелом состоянии несоответствие между общим, альбумин-скорректированным и ионизированным кальцием может быть частично объяснено гипоальбуминемией и/или метаболическим ацидозом (табл. 1). Залога и соавт. проанализировали, насколько ионизированный кальций, рассчитанный по номограмме МакЛина-Гастингса совпадает с ионизированным кальцием у больных в реанимации после хирургических вмешательств [80]. Авторы пришли к выводу, что из 156 пациентов только у 12% имелась истинная гипокальциемия по измеренному ионизированному кальцию, тогда как 71% и 58% соответственно, была по общему и расчетному ионизированному кальцию. В подобных случаях для выявления гиперкальциемии или гипокальциемии рекомендуется прямое измерение ионизированного кальция [80].

В нескольких работах изучалась точность определения уровня общего кальция и его скорректированного значения для прогнозирования гипокальциемии или гиперкальциемии у больных в тяжелом состоянии. В работе Зулкуфи и соавт. проанализировали результаты 281 пациента, госпитализированного в тяжелом состоянии в ОРИТ. По результатам, альбумин-скорректированный кальций не являлся оптимальным показателем в данной когорте больных и показал значительные разногласия с концентрацией ионизированного кальция [8]. Согласно результатам исследования, нормокальциемия по результатам расчета кальция, скорректированного на альбумин, наблюдалась у 60,9% пациентов, тогда как определение уровня ионизированного кальция выявило гипокальциемию у 92,2% больных. Коррекция кальция на альбумин по формуле Пейна неверно классифицировала статус кальция у 66,4% пациентов с гипокальциемией, отнеся к категории нормальных или высоких значений скорректированного уровня кальция; общая точность составила 37,7%. Опубликованные в литературе уравнения для расчета скорректированного кальция у пациентов в критическом состоянии продемонстрировали более низкую точность в диапазоне от 19,9 до 37%. На момент забора крови 65,1% пациентов находились на искусственной вентиляции легких, 18,1% — на гемодиализе и 37,4% переливались компоненты крови в предшествующие 24 часа, однако статистически значимой связи между перечисленными клиническими состояниями и измеренным уровнем ионизированного кальция не наблюдалось [8]. В исследовании Дикерсон и соавт. при обследовании 100 пациентов после политравмы, находящихся в отделении интенсивной терапии, в 27% случаях отмечалась гипо- или гиперкальциемия по ионизированному кальцию [81]. При сравнении ионизированного кальция со скорректированным кальцием по опубликованным в литературе 22 алгоритмам было обнаружено, что все эти алгоритмы обладают низкой чувствительностью и дают очень высокую долю ложноотрицательных результатов (75%±32%), и авторы также рекомендуют измерять ионизированный кальций у больных в тяжелом состоянии [81]. Схожие данные получены в исследованиях Сломп и соавт. [82] и Бирнс и соавт. [83].

Пфитзенмайер и соавт. в 2007 г. предложили новую формулу для расчета ионизированного кальция у больных в возрасте старше 80 лет и с уровнем альбумина менее 35 г/л [84]:

Ионизированный кальций (ммоль/л) = 0,592 – 0,00449 х белок (г/л) + 0,410 х общий кальций (ммоль/л)

Однако Бйоркман и соавт. исследовали уровень ионизированного кальция и кальция, скорректированного на альбумин, по формуле Пейна и на общий белок по формуле Пфитзенмайера у тяжелобольных пожилых пациентов и в общей популяции людей старшего возраста и пришли к выводу, что оба суррогатных показателя плохо предсказывают истинную кальциемию в этих группах [85].

В совокупности полученные результаты позволяют сделать вывод, что кальций, скорректированный на альбумин, не является оптимальным показателем концентрации ионизированного кальция у больных в тяжелом состоянии и пожилых больных. Ионизированный кальций рекомендуется определять пациентам с подозрением на гипокальциемию, а полученные результаты следует интерпретировать вместе с показателями рН крови.

## Гиперкальциемия

ПГПТ и злокачественные новообразования являются двумя самыми частыми причинами гиперкальциемии [2]. Гленденнинг и соавт. в ретроспективном исследовании 60 гистологически верифицированных случаев ПГПТ показали, что у 22% имелся нормальный уровень альбумин-скорректированного кальция, тогда как у 98% имелась гиперкальциемия по ионизированному кальцию [86]. В ретроспективном исследовании Онг и соавт., включившем 5490 пациентов, у 927 больных концентрация ионизированного кальция и/или общего кальция выходила за пределы референсного интервала [87]. В целом диагностическая дискордантность при классификации статуса кальция наблюдалась у 692 (12,6%) из 5490 пациентов. При определении только общего кальция было пропущено 156 (45%) из 346 случаев гиперкальциемии по ионизированной фракции. В когорте с ПТГ-зависимой гиперкальциемией у 130 (41%) из 315 больных на момент постановки диагноза наблюдалась изолированная гиперкальциемия по уровню ионизированного кальция. Среди 143 пациентов с гистологически верифицированной гиперплазией, аденомой или карциномой околощитовидной железы у 34 больных (24%) на момент постановки диагноза выявлено изолированное повышение уровня ионизированного кальция. Примечательно, что эти пациенты были моложе (р=0,022), имели более низкие концентрации ПТГ (р≤0,001) и кальция, скорректированного на альбумин (р≤0,001), а также лучшую функцию почек (р≤0,001) в сравнении с пациентами с одновременным повышением общего и ионизированного кальция. Кроме того, у больных с солитарными аденомами и высокими значениями обеих фракций кальция средний вес удаленной околощитовидной железы был выше, чем у пациентов с изолированным повышением ионизированного кальция (0,945 г против 0,521 г, р=0,006) [87].

В проспективном исследовании Рианчо и соавт. исследовали показатели кальция крови у 188 пациентов с различными злокачественными заболеваниями, при этом около 50% имели отдаленные метастазы, из них у 17% — в кости [88]. Корреляция общего кальция с ионизированным кальцием не улучшалась при использовании нескольких корректировочных формул, в том числе формулы Пейна. В 57% случаев общий кальций не смог предсказать гиперкальциемию по уровню ионизированного кальция, однако авторы делают вывод, что, поскольку умеренная гиперкальциемия не была ассоциирована с плохим прогнозом по сравнению с выраженной гиперкальциемией, измерение ионизированного кальция у таких больных нецелесообразно [88]. В проспективном исследовании Иджаз и соавт. исследовали уровень ионизированного, общего и альбумин-скорректированного кальция у 97 пациентов с различными онкологическими заболеваниями и у 39 здоровых лиц, подобранных по полу и возрасту [89]. Гиперкальциемия злокачественных заболеваний была зафиксирована в 38% случаев по ионизированному кальцию, что было значимо выше по сравнению с общим кальцием (8,4%) и альбумин-скорректированным кальцием (10,6%) (р<0,001) [89].

Таким образом, для диагностики гиперкальциемических состояний определение ионизированного кальция более информативно, по сравнению с исследованием общего кальция и с поправкой на альбумин.

## ЗАКЛЮЧЕНИЕ

Единственная цель, заложенная в концепцию определения альбумин-скорректированного кальция, — сделать так, чтобы этот показатель лучше отражал концентрацию ионизированного кальция, то есть расчет некоего суррогатного маркера, который можно использовать вместо прямого измерения ионизированного кальция. В настоящее время существуют противоречия между основными рекомендациями по различным нарушениям фосфорно-кальциевого обмена, в которых рекомендуется корректировать уровень общего кальция на альбумин, и большим количеством исследований, доказывающих нецелесообразность такой коррекции. Среди ключевых спорных моментов можно перечислить следующие. Практикующий врач, вводя показатели альбумина и общего кальция в калькуляторы, размещенные в сети Интернет, зачастую не знает, какая именно формула заложена в основу калькулятора, а также не знает, какими методами измерялись общий кальций и альбумин, особенно если пациент пришел на амбулаторный прием. Так, формула Пейна разрабатывалась при определении альбумина методом БКЗ, тогда как в настоящее время в лабораториях также используется метод БКП, что влияет на результат определения альбумина. Также различные лаборатории могут предоставлять различные референсные интервалы для общего кальция и для альбумина, и использование верхней границы референсного интервала общего кальция для альбумин-скорректированного кальция вызывает сомнение. В приведенных выше исследованиях утверждается, что разработанные формулы могут быть применимы только к определенным когортам больных [44], или только для определенных методов исследования [34], что каждая лаборатория должны разрабатывать свое уравнение регрессии [26]. Некоторые исследователи выдвинули гипотезу, согласно которой формулы корректировки основаны на неправильно построенных регрессионных моделях. Согласно мнению этих ученых, существующие формулы в основном рассчитаны методом простой линейной регрессии и не предполагают поправку на другие переменные кроме альбумина [76].

Интересен вопрос, в каких случаях предполагается корректировать общий кальций на альбумин: нужно ли это делать во всех случаях, в том числе и при нормальном уровне общего кальция в крови, или только при отклонениях от референсного интервала уровня кальция или уровня альбумина? В большинстве приведенных выше исследований авторы приходят к выводу, что такая коррекция нецелесообразна. В случае гипоальбуминемии коррекция будет искажать результат [63][75], хотя в рекомендациях по ХБП рекомендуют корректировать кальций именно у больных с гипоальбуминемией. При гипоальбуминемии может быть снижен уровень общего кальция, при этом уровень ионизированного кальция остается в норме. Цель корректировочных формул как раз выявить это несоответствие, однако при гипоальбуминемии формулы хуже предсказывают уровень ионизированного кальция [90]. Из недостатков некоторых исследований можно перечислить ретроспективный дизайн, не позволяющий оценить качество исследования ионизированного кальция, смешение выборок пациентов из отделений реанимаций и других отделений [63][74], а также смешение амбулаторных и стационарных пациентов [62][76]. Не во всех исследованиях указаны чувствительность, специфичность и отношения правдоподобия для общего и альбумин-скорректированного кальция при гипо- и гиперкальциемии.

В 2015 г. Ассоциацией клинической биохимии и лабораторной медицины (Великобритания) была предпринята попытка создания проекта консенсуса о коррекции кальция на альбумин [91]. Было сформулировано десять рекомендаций, включая необходимость систематического обзора литературы по данной проблеме, необходимость каждой лаборатории разрабатывать свое уравнение регрессии в соответствии с используемыми методами определения кальция и альбумина, а не использовать формулы из литературы, и периодически проверять достоверность своей формулы, а также необходимость для лабораторий, предоставляющих результат альбумин-скорректированного кальция, наличие внешнего контроля качества [91]. В целом в работе ставится больше вопросов, на которые необходимо ответить в будущем, а из ее недостатков можно отметить слишком небольшое количество источников литературы (всего 11) [91].

Таким образом, при анализе вышеперечисленных исследований можно сделать вывод, что определение ионизированного кальция более информативно по сравнению с исследованием общего кальция при некоторых патологических состояниях, связанных с изменением содержания белков и pH крови, при обследовании пациентов с заболеваниями почек, а также для диагностики гиперкальциемических состояний. Диагностическая точность широко используемых корректировочных формул в некоторых клинических ситуациях может быть даже хуже диагностической точности нескорректированного общего кальция. Применение формул коррекции у пациентов с гипоальбуминемией может привести к завышению уровня кальция. Возможно, целесообразно верифицировать формулы коррекции общего кальция на альбумин в российской популяции на базе лабораторий ведущих федеральных центров. При наличии подозрения на нарушения обмена кальция и при возникновении сомнений в точности общего кальция целесообразно измерять уровень ионизированного кальция с соблюдением всех преаналитических и аналитических правил.

## ДОПОЛНИТЕЛЬНАЯ ИНФОРМАЦИЯ

Источники финансирования. Работа выполнена в рамках государственного задания «Новые технологии диагностики и дифференциальной диагностики первичного и вторичного остеопороза на фоне эндокринопатий и орфанных заболеваний скелета» № 124020700097-8.

Конфликт интересов. Авторы декларируют отсутствие явных и потенциальных конфликтов интересов, связанных с содержанием настоящей статьи.

Участие авторов. Все авторы одобрили финальную версию статьи перед публикацией, выразили согласие нести ответственность за все аспекты работы, подразумевающую надлежащее изучение и решение вопросов, связанных с точностью или добросовестностью любой части работы.
